# Synthesis, X-ray Structures, and Optical and Magnetic Properties of Cu(II) Octafluoro-octakisperfluoro(isopropyl)phthalocyanine: The Effects of Electron Addition and Fluorine Accretion

**DOI:** 10.1021/acs.inorgchem.3c00887

**Published:** 2023-07-12

**Authors:** Maxim A. Faraonov, Ilya A. Yakushev, Evgeniya I. Yudanova, Marius Pelmus, Sergiu M. Gorun, Akihiro Otsuka, Hideki Yamochi, Hiroshi Kitagawa, Dmitri V. Konarev

**Affiliations:** † Federal Research Center of Problems of Chemical Physics and Medical Chemistry, Russian Academy of Sciences, Moscow Region, Chernogolovka 142432, Russia; ‡ Kurnakov Institute of General and Inorganic Chemistry, Russian Academy of Sciences, Moscow 119991, Russia; § Department of Chemistry and Biochemistry and Center for Functional Materials, Seton Hall University, South Orange, New Jersey 07079, United States; ∥ Division of Chemistry, Graduate School of Science, Kyoto University, Sakyo-ku, Kyoto 606-8502, Japan

## Abstract

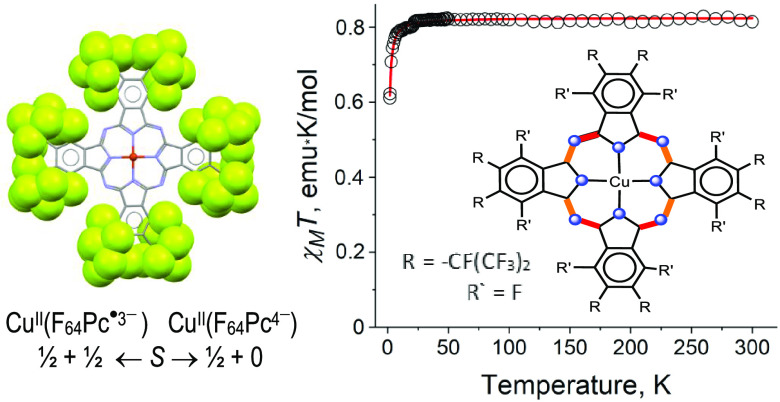

The stepwise reduction of copper(II) 1,4,8,11,15,18,22,25-octafluoro-2,3,9,10,16,17,23,24-octakisperfluoro(isopropyl) phthalocyanine (Cu^II^F_64_Pc) in *o*-dichlorobenzene (C_6_H_4_Cl_2_) by potassium graphite in the presence of cryptand(K^+^), abbreviated L^+^, results in the formation of (L^+^)[Cu^II^(F_64_Pc^•3–^)]^−^·2C_6_H_4_Cl_2_ (**1**), (L^+^)_2_[Cu^II^(F_64_Pc^4–^)]^2–^·C_6_H_4_Cl_2_ (**2**), and (L^+^)_2_[Cu^II^(F_64_Pc^4–^)]^2–^ (**3**) complexes. Single-crystal X-ray structures revealed their composition and a monotonic increase with increased phthalocyanine (Pc) negative charges of the magnitude of alternative shortening and elongation of the prior equivalent N_meso_–C bonds. The complexes are separated by bulky *i-*C_3_F_7_ substituents, large cryptand counterions, and solvent molecules. Weak, new bands are generated in the visible and near-infrared (NIR) domains upon reductions. The one-electron reduced complex, [Cu^II^(F_64_Pc^•3–^)]^−^, is a diradical, exhibiting broad electron paramagnetic resonance (EPR) signals, with intermediate parameters between those typical to Cu^II^ and F_64_Pc^•3–^. The two-electron reduced complexes, [Cu^II^(F_64_Pc^4–^)]^2–^, contain a diamagnetic F_64_Pc^4–^ macrocycle and a single spin, *S* = 1/2, on Cu^II^. The bulky perfluoroisopropyl groups are suppressing intermolecular π–π interactions between Pcs in the [Cu^II^(F_64_Pc^*n*–^)]^(*n*−2)–^ (*n* = 3, 4) anions, **1**–**3**, similar to the case of the nonreduced complex. However, π–π interactions between **1** and *o*-dichlorobenzene are observed. The d^9^ and Pc electrons in **1** are antiferromagnetically coupled, *J* = −0.56 cm^–1^, as revealed by superconducting quantum interference device (SQUID) magnetometry, but the coupling is at least 1 order of magnitude smaller compared with the coupling observed for Cu^II^(F_8_Pc^•3–^) and Cu^II^(F_16_Pc^•3–^), a testimony to the F accretion effect of rendering the Pc macrocycle progressively more electron-deficient. The data for Cu^II^(F_64_Pc) provide structural, spectroscopic, and magnetochemical insights, which establish a trend of the effects of fluorine and charge variations of fluorinated Pcs within the macrocycle series Cu^II^(F*_x_*Pc), *x* = 8, 16, 64. Diamagnetic Pcs might be useful for photodynamic therapy (PDT) and related biomedical applications, while the solvent-processable biradicalic nature of the monoanion salts may constitute the basis for designing robust, air-stable electronic, and magnetically condensed materials.

## Abstract 2

Salts of reduced electron-deficient perfluoro phthalocyanine 19 and 20 π-electron complexes are air-stable, exhibit alternate N_meso_−C bond lengths, and the metal- and ligand-centered unpaired electrons are intramolecularly coupled antiferromagnetically.

## Introduction

The phthalocyanines (Pcs) are a large family of macrocycle compounds used as industrial dyes, currently investigated as materials for optical, electronic, and photoelectronic devices, catalysts, phototherapeutic drugs, and other biomedical applications.^1−10^ Their properties can be effectively and rationally tuned by variations of the central atoms, axial ligands, and macrocycle substituents to yield both electron-rich and electron-poor macrocycles with potential wide and diverse applications. The introduction of strong electron-withdrawing substituents leads to a positive shift of the Pcs reduction potentials stabilizing their anionic species, including in air.^11^ The largest redox shift has been achieved to date by the introduction of eight, bulky perfluoro(isopropyl) (*i*-C_3_F_7_) groups at the peripheral positions of perfluoro Pcs, forming 1,4,8,11,15,18,22,25-octafluoro-2,3,9,10,16,17,23,24-octakisperfluoro(isopropyl)phthalocyanine, present in MF_64_Pc, M = Cu^II^, Co^II^, Zn^II^, V^IV^O, etc. The complexes enhanced solubility in organic solvents, and chemical stability rendered them suitable for applications in photodynamic therapy,^12,13^ catalysis,^14−18^ and materials for electrochromic displays.^19−22^ Their strong electron acceptor ability allows the reversible addition of 1 or 2 electrons to form [M(F_64_Pc^•3–^)]^−^ or [M(F_64_Pc^4–^)]^2–^, while electron loss is suppressed.^13,19,20,23^ Reduction or oxidation of Pcs yield species with an unpaired electron delocalized over the macrocycles. This electron can participate in the magnetic coupling of spins or high conductivity.^24,25^ Moreover, in the case of paramagnetic central metal atoms, an unpaired electron localized on metal can interact with the conducting electron delocalized over the macrocycles leading to giant magnetoresistance.^26,27^ Generally, metal phthalocyanine anions are very air-sensitive due to strongly negative reduction potentials^11^ but that is not true for the air-stable radical trianions F_64_Pc^•3–^. Therefore, the [M(F_64_Pc^•3–^)]^−^ species can serve as components in magnetic, conducting, magneto-, and electro-optical devices working in air. Paramagnetic ions, such as Cu(II), are of special interest for electrochemically coupled magnetic interactions since copper(II) complexes have two magnetic centers in monoanionic species, viz. [Cu^II^(macrocycle)^•3–^]^−^.^28−31^

The effect of fluorination as a tool for the rational design of new materials with predictable structural, spectroscopic, magnetic properties, and reactivity is of interest.

We report here the stepwise 1- and 2-electron reductions of Cu^II^(F_64_Pc) to form anions, crystal growth, and structural characterization of the anions complexed with potassium cryptand counterions, the optical and magnetic properties of the new materials, and resulting trends imparted by electron addition and fluorine accretion.

## Results and Discussion

**Figure 1:**
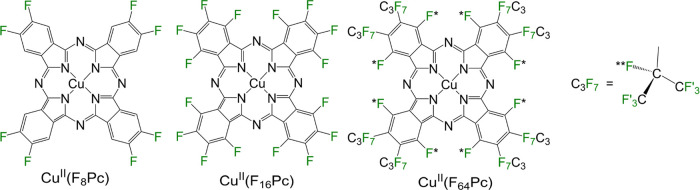
Structures of F*_x_*Pc^*n*–^ copper complexes. The degree and type of fluorination range from half-aromatic F, *x* = 8, to full aromatic F, *x* = 16, to half-perfluoroalkyl and half-aromatic F, *x* = 64.

The structures of F*_x_*Pc^*n*–^ copper complexes are shown in Figure 1, *x* = 8, 16, and 64 and *n* = 2, 3, and 4.

The fluorine atoms are labeled separately based on their positions in the molecule. As shown in Figure 1, for *x* = 8, only the peripheral type F is present; 2 types are present for *x* = 16: peripheral and nonperipheral; and 3 types of F are present for *x* = 64: primary (labeled with ′), tertiary F (labeled with **), and aromatic, nonperipheral (labeled with *).

The chemical composition of the complexes with *x* = 64 is shown in Table 1. For complexes **1**–**3**, reported here, the counterions and solvated molecules are also shown, as revealed by their single-crystal X-ray structures (see below). The reduction degree of the ligand, relative to F_64_Pc^2–^, is also shown.

**Table 1:** Chemical Composition of Cu(II) Complexes of the F_64_Pc Ligand^a^

Label	Formula, X-ray crystals	F_64_Pc^2–^ extra charge
**0**	[Cu^II^(F_64_Pc^2–^)(C_4_H_8_O_2_)_2_]	0
**1**	{cryptand(K^+^)}[Cu^II^(F_64_Pc^•3–^)]^−^·2C_6_H_4_Cl_2_	–1
**2**	{cryptand(K^+^)}_2_[Cu^II^(F_64_Pc^4–^)]^2–^·C_6_H_4_Cl_2_	–2
**3**	{cryptand(K^+^)}_2_[Cu^II^(F_64_Pc^4–^)]^2–^	–2

a Cryptand: 4,7,13,16,21,24-hexaoxa-1,10-diazabicyclo[8.8.8]hexacosane; C_4_H_8_O_2_: ethyl acetate; C_6_H_4_Cl_2_: *o*-dichlorobenzene.

Anionic salts **1**–**3** were prepared by reducing the parent complex, [Cu^II^(F_64_Pc^2–^)], **0**, with potassium graphite (KC_8_) in *o*-dichlorobenzene in the presence of 1 or 2 equiv of 4,7,13,16,21,24-hexaoxa-1,10-diazabicyclo[8.8.8]hexacosane cryptand. The previously reported reductions of Cu^II^(F*_x_*Pc) to [Cu^II^(F*_x_*Pc)^•3–^]^−^ and [Cu^II^(F*_x_*Pc)^4–^]^2–^ (*x* = 8, 16)^28,29^ were performed with sodium fluorenone ketyl, *E*_redox_ = −1.30 V vs saturated calomel electrode (SCE),^32^ or with sodium cyclopentadienyl cobalt dicarbonyl (Na^+^)[CpCo(CO)_2_]^−^, *E*_redox_ = −1.85 V vs SCE, also in the presence of the cryptand.^29^ For *x* = 64, KC_8_ afforded the selective reduction of [Cu^II^(F_64_Pc^2–^)] by 1 or 2 electrons. The anions are paired with cryptand(K^+^) cations. The monoreduced complex **1** is deep-blue in *o*-dichlorobenzene, yielding large, black X-ray quality prismatic crystals with a characteristic copper luster by the slow diffusion of *n*-hexane over a period of 1–2 months. The composition of **1** was determined by X-ray diffraction and was supported by elemental analysis (see the Experimental Section). The reduction of **0** by two equivalents of cryptand affords the isolation of **3** as small, black, elongated plates, but only in extremely low yield. These crystals belong only to a single crystal phase, and here we present only the crystal structure of **3**. A different strategy was used to obtain complex **2**. Thus, one equivalent of cryptand and KC_8_ was added to the solution that generated **1**, and the stirring continued for 24 h giving a red-violet solution, from which **2** could be isolated as a crystalline material in a small yield after 1 month of diffusion of *n*-hexane. We determined the crystal structure of **2** and studied its electron paramagnetic resonance (EPR) and optical properties using several single crystals, which were tested by X-ray diffraction and were shown to have the same unit cell parameters as **2**. Compositions of **2** and **3** were determined by X-ray diffraction. The continuation of the titration with a third equivalent of cryptand and KC_8_ yielded a blue-pink solution, but no crystalline products could be isolated in this case.

These results are consistent with the following rationalization. Metal phthalocyanines are relatively weak electron acceptors, but their ability to accept electrons can be increased by the introduction of electron-withdrawing substituents. The substitution of aromatic hydrogen in H_16_Pc with fluorine indeed enhances the electronic-accepting capability of the Pc, as well as its thermal and chemical robustness, beneficial properties for catalytic applications. The formal replacement of the eight peripheral F atoms of F_16_PcCu with *iso*-C_3_F_7_-groups, to give F_64_PcCu, shifts the redox potentials of Cu^II^F*_x_*Pc toward even more positive values.^13,19,20,23^ A positive shift of about 0.4 V is observed for the F_64_Pc scaffold vs F_16_Pc. Notably, the positive shift is metal-independent^34^ since the frontier orbitals consist of an atomic orbital of the ligand.

Naturally, the ligand-based frontier orbitals are susceptible to variations in ligand substituents. The perfluoroalkyl substituents exert a higher inductive effect compared with the aromatic F atoms, as the positive dipole of CF_3_ groups creates positive potential on adjacent atoms.^35^ Such a shift relative to the unsubstituted Pc is even higher, being about 0.7 V.^19^

Anions derived from Cu^II^(F_64_Pc) exhibit a relatively enhanced solubility in organic solvents vs complexes with *x* < 64 due to the presence of bulky perfluoroisopropyl substituents. The (F_64_Pc)^•3–^ radical trianion can be generated electrochemically, chemically using reducing agents, and by photochemical reduction using hydrazine hydrate (*E*_redox_ = – 0.01 V vs SCE^36^) in dimethylformamide (DMF)^33^ or in ethanol even without any added reductant.^16^ Taken together, the results suggest that F_64_Pc is the strongest electron acceptor fluorinated phthalocyanine macrocycle reported to date.

The X-ray structures of the reduced species reveal the variations in molecular parameters induced by the addition of more electrons.

## X-ray Crystal Structures

**Figure 2:**
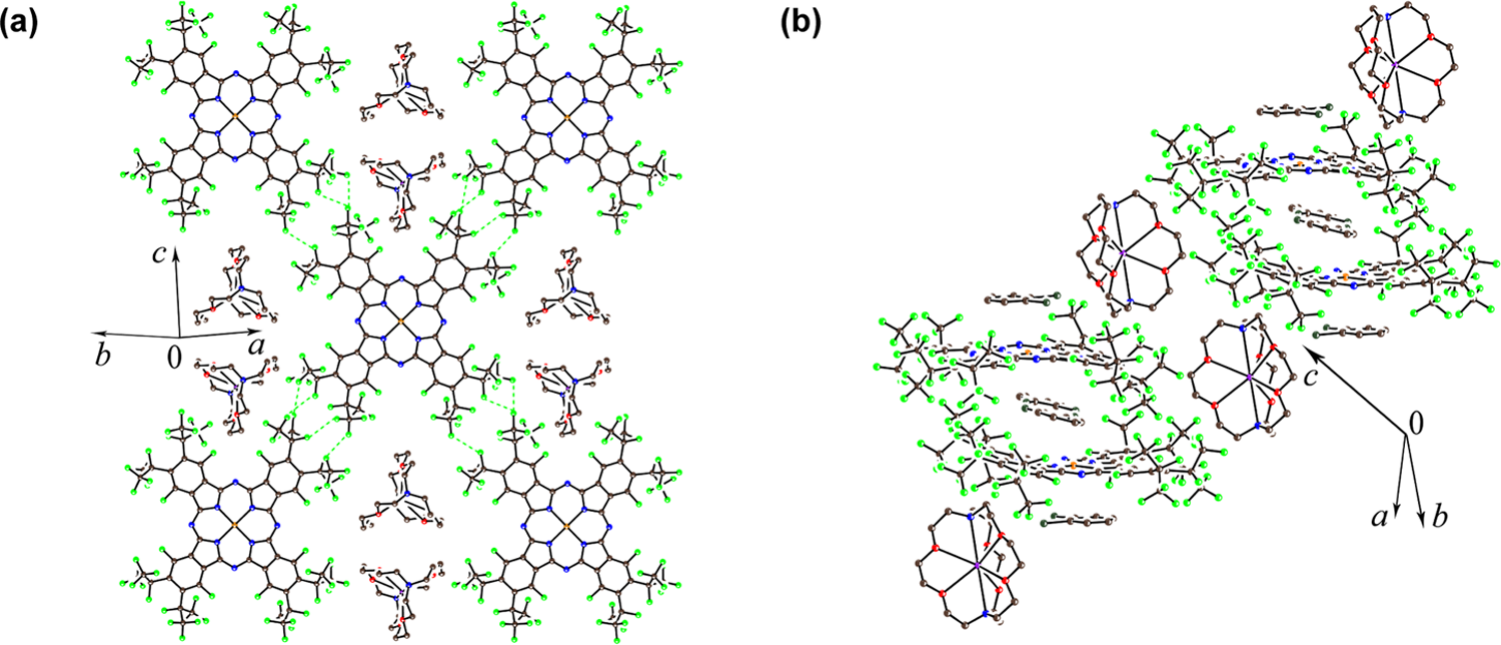
X-ray structure of **1**. Short van der Waals contacts are shown by green, dashed lines. (a) View, in projection, perpendicular to the Pc layers, showing the cations, but omitting the solvents for the sake of clarity. (b) View, approximately parallel to the Pc layers, showing both the cations and the intercalated *o*-dichlorobenzene solvent. Additional views are presented in Figure S1a–c, Supporting Information.

**Figure 3:**
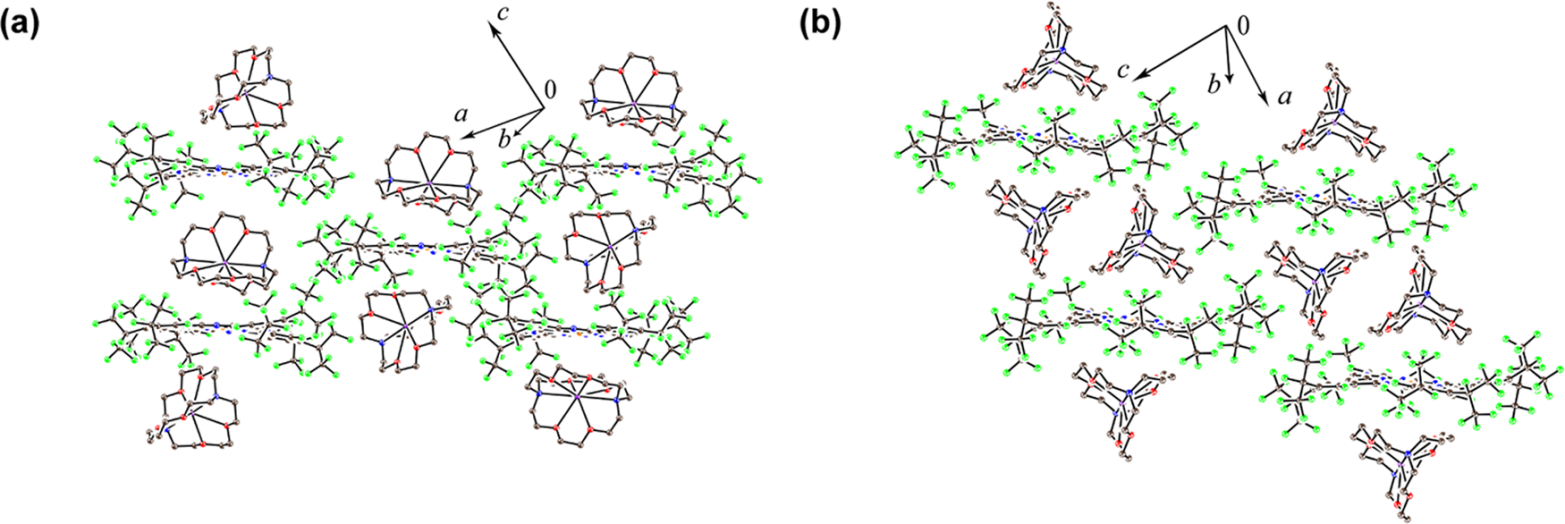
Crystal structures of complexes containing {cryptand(K^+^)}_2_[Cu^II^(F_64_Pc^4–^)]^2–^. The viewing direction is approximately parallel to the Pc layers. The *o*-dichlorobenzene solvent in **2** has been omitted. Additional views are presented in Figure S1d–f for **2** and Figure S1g–i for **3**. (a) {cryptand(K^+^)}_2_[Cu^II^(F_64_Pc^4–^)]^2–^·C_6_H_4_Cl_2_, **2**. (b) {cryptand(K^+^)}_2_[Cu^II^(F_64_Pc^4–^)]^2–^, **3**.

Two different views of the crystal structure of **1**, {cryptand(K^+^)}[Cu^II^(F_64_Pc)^•3–^]^−^·2C_6_H_4_Cl_2_, are shown in Figure 2, while the structures of {cryptand(K^+^)}_2_[Cu^II^(F_64_Pc^4–^)]^2–^, crystallized with solvent, **2**, and without it, **3**, are shown in Figure 3. The packing diagrams of two unit cells of **1**–**3**, viewed along the crystallographic axes, are shown in Figure S1, Supporting Information.

The X-ray structures unambiguously determine the compositions listed in Table 1. Monoanions of Cu^II^(F_64_Pc) are present in **1**, whereas dianions are present in **2** and **3**. The overall shape of the Pcs is nonplanar, with some twisting of benzene rings by about 17° in **1** and a slight doming of the macrocycle. The departure of Pc from planarity, combined with the steric hindrance of the peripheral *iso*-alkyl substituents, impacts molecular packing (see also Figure S1).

The related [Cu^II^(F*_x_*Pc^•3–^)]^−^ and [Cu^II^(F*_x_*Pc)^4–^]^2–^ anions (*x* = 8, 16), with the same charges as the anions with *x* = 64 but with only aromatic F substituents tend to form closely packed architectures and π-stacks, or chains with π–π interactions, features that affect their optical and magnetic properties.^28,29^ A different situation is observed for **1**–**3**, based on [Cu^II^(F_64_Pc)^*n*−^]^(*n*−2)–^ (*n* = 3, 4) anions.

The [Cu^II^(F_64_Pc)^•3–^]^−^ anions in **1** form chessboard-like layers in which macrocycles alternate with the cryptand(K^+^) cations (Figure 2a). Short van der Waals F (C_3_F_7_)···F (C_3_F_7_) contacts of 2.6–2.9 Å are present, indicating a contribution to packing. No π–π interactions between Pc cores are present in **1**.

**Figure 4:**
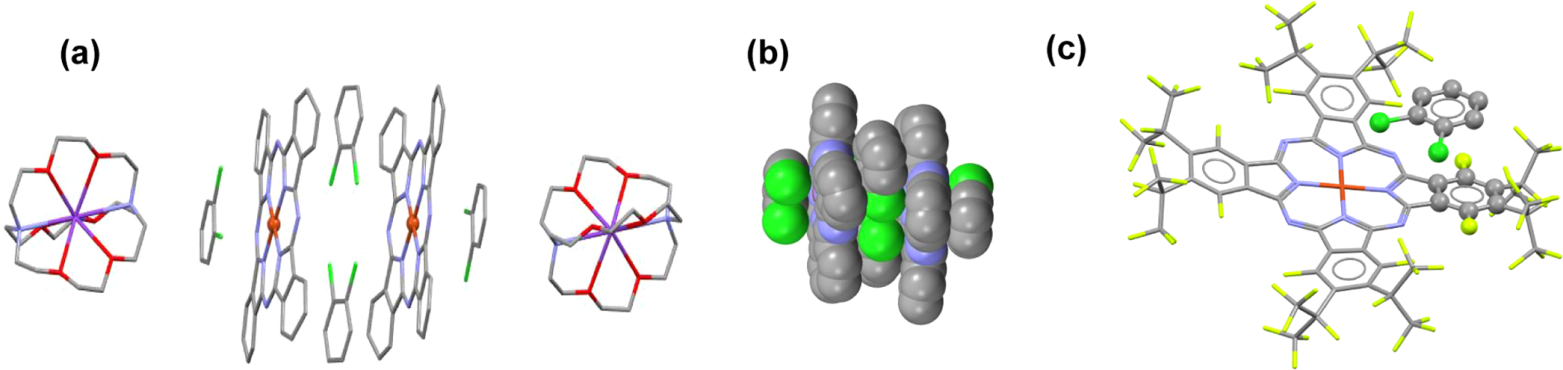
Simplified view of the aromatic solvent interactions with the Pc in **1**. (a) The assembly of the cations, Pc, and *o*-dichlorobenzene located within the “bivalve”, shell-like cavity formed by the concave sides of Pcs, termed ″interior″ solvents, as well as on the convex sides, termed ″exterior″ solvents, are shown. The distances between the ″exterior″ solvents and the Pc coordination plane reach a low 3.25 Å, which is shorter than the 3.35 Å value for the graphite crystal interplanar distance, but this interaction did not result in its identification by the aromaticity test as π-stacking.^39^ Hydrogens and Pc substituents have been omitted for the sake of clarity. (b) Space-filling representation of the assembly of panel (a) but with cations and Pc substituents omitted for the sake of clarity. (c) Ball-and-stick representations of one of the two aromatic interactions between an ″interior″ *o*-dichlorobenzene and a benzene ring of the Pc. The stacking, ∼3.9 Å, is termed as “strong”, reaching about ∼90% of the maximum strength^39^ (see also Figure S5 and Table S1 for the complete list of strong interactions).

The Pc layers are separated by bulky cryptand(K^+^) cations and *o*-dichlorobenzene molecules (Figure 2b). Unlike **1**, the [Cu^II^(F_64_Pc^4–^)]^2–^ dianions are isolated (noninteracting) in **2** and **3**. The large voids are occupied by cryptand(K^+^) cations. Note that molecular aggregations are suppressed by *iso-*C_3_F_7_ groups irrespective of the metal center of M(F_64_Pc), viz. M = Cu^II^, Co^II^, Zn^II^, and V^IV^O.^13,17,23,38^ Interestingly, the intermolecular interactions of {cryptand(K^+^)}_2_[Cu^II^(F_64_Pc^4–^)]^2–^ appear sufficiently plastic to allow its crystallization with and without solvent, **2** and **3**, respectively, but the solvent ability to participate in π–π interactions with Pcs may play a role in packing. Indeed, π-type interactions are detected in **1** (Figure 4c) between the solvents, nested in the concave sides of the Pcs, and the benzene rings of the Pcs (Figures 4a and 2b). Such interactions are not present in **2**.

The stable, 18 π-electron aromaticity is largely lost by the formation of less stable 19 and 20 π-electron systems. The addition of electrons, combined with fluorinated substituent accretion, has structural consequences. A comparison of geometric features of **0**, **1**, **2**, and **3** reveals these effects (Table 2).

**Table 2:** Bond Lengths Observed via X-ray Diffraction in Fluorinated Copper Phthalocyanines^a^

		Average bond lengths, Å			
Compound^reference^	Charge state of the Pc	Cu–N_pyr_	C–N_pyr_	C–N_meso_ Δshort/long	C–F average
Cu^II^(F_8_Pc^2–^)^40^	–2	1.939(5)	1.375(8)	1.313(8)/1.341(8)	1.352(7)
				0.028	
{cryptand(Na^+^)}[Cu^II^(F_8_Pc^•3–^)]^−^ ^29^	–3	1.9536(17)	1.377(3)	1.312(3)/1.352(3)	1.360(2)
				0.040	
{Bu_4_N^+^}_2_[Cu^II^(F_8_Pc^4–^)]^2–^·2C_6_H_4_Cl_2_^28^	–4	1.961(5)	1.386(8)	1.283(8)/1.374(8)	1.381(7)
				0.091	
Cu^II^(F_16_Pc^2–^)^41^	–2	1.952(2)	1.378(3)	1.320(3)/1.323(2)	1.346(2)
				0.003	1.341(2)
[Cu^II^(F_16_Pc^•3–^)]^–^ in (PPN^+^)_3_[Cu(F_16_Pc)]_3_^3–^·2C_6_H_5_CN^29^	–3	1.954(2)	1.377(2)	1.325(2)/1.328(2)	1.345(2)
				0.003	
[Cu^I^(F_16_Pc^**2**–^)]^−^ in (PPN^+^)_3_[Cu(F_16_Pc)]_3_^3–^·2C_6_H_5_CN^29^	–2	1.955(2)	1.378(2)	1.326(2)/1.329(2)	1.345(2)
				0.003	
{cryptand(Na^+^)}_2_[Cu^II^(F_16_Pc^4–^)]^2–^ ^28^	–4	1.954(2)	1.383(2)	1.305(2)/1.363(2)	1.348(2)
				0.058	
Cu^II^(F_64_Pc^2–^)·2C_4_H_8_O_2_ (**0**) ^23^	–2	1.958(3)	1.365(5)	1.326(5)/1.324(5)	1.344(5)*
				0.002	1.370(5)**
					1.331(5)′
{cryptand(K^+^)}[Cu^II^(F_64_Pc^•3–^)]^−^·2C_6_H_4_Cl_2_ (**1**) this work	–3	1.948(3)	1.375(5)	1.333(5)/1.319(5)	1.343(5)*
				0.014	1.368(5)**
					1.330(5)′
{cryptand(K^+^)}_2_[Cu^II^(F_64_Pc^4–^)]^2–^·C_6_H_4_Cl_2_ (**2**) this work	–4	1.954(3)	1.382(4)	1.363(5)/1.297(5)	1.344(5)*
				0.066	1.367(5)**
					1.331(6)′
{cryptand(K^+^)}_2_[Cu^II^(F_64_Pc^4–^)]^2–^ (**3**) this work	–4	1.956(2)	1.382(2)	1.370(2)/1.293(2)	1.342(2)*
				0.077	1.370(2)**
					1.323(2)′

a *, **, and ′ show C–F bonds with different fluorine atoms in the F_64_Pc macrocycle (as shown in Figure 1).

**Figure 5:**
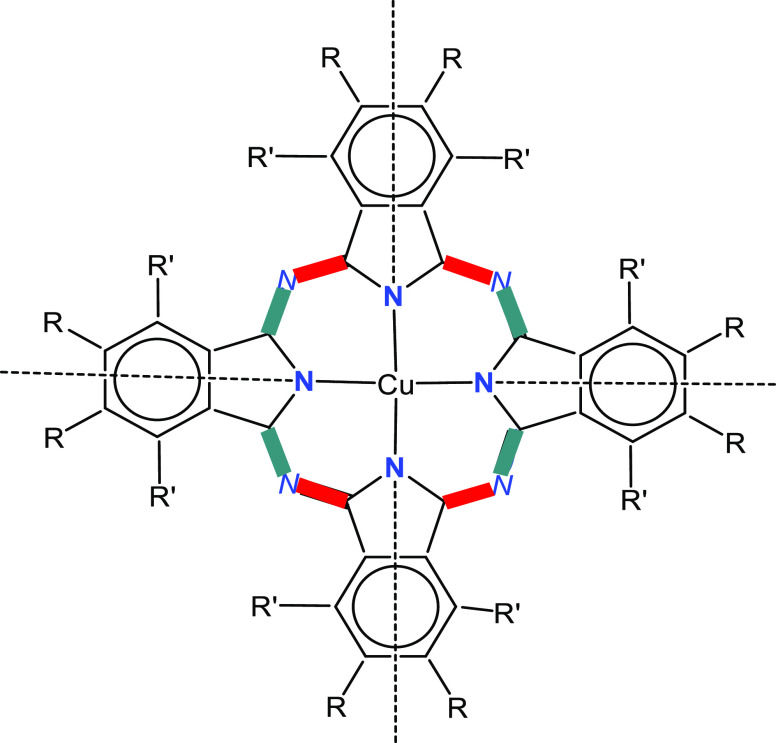
Schematic illustration of the distortions listed in Table 2. *N* and **N** are the meso and pyrrolic N atoms, respectively. R = F or C_3_F_7_. R′ = H or F. Bond elongation and shortening, relative to the parent Cu^II^(F_64_Pc^2–^)·2C_4_H_8_O_2_, are shown as red and green bars, respectively. The dotted lines mark the location of mirror and *C*_2_ symmetry elements. Although the pyrrolic C–N bonds are shown as single, their observed lengths are statistically indistinguishable, suggesting sp^2^ delocalization.

Six coordination via axial solvent binding appears common for crystals of (F_64_Pc) M = Cu^II^, Co^II^, Zn^II^, V^IV^O.^13,17,23,34^ A reduced, four-coordination, square-planar geometry is observed for **1**–**3**, the copper(II) ions being located almost exactly in the center of the average plane formed by the four N_pyr_ atoms. The Cu displacement from this plane is only 0.014 Å for **1**, 0.022 Å for **2**, but 0.000 for **3**. The Cu–N bond distances do not vary significantly, despite the fact that the Cu is six-coordinated in **0** but four-coordinated in **1**–**3**. The most statistically significant changes are observed for the lengths of the N_meso_–C bonds.^30,31,42−45^ Shorter and longer N_meso_–C bonds, relative to the parent macrocycle, Cu^II^(F_64_Pc), for which the bonds are the same, are present in the reduced macrocycles with *x* = 8, 16. The common pattern is illustrated in Figure 5.

The fourfold rotational axis of the nonreduced molecule is reduced to a twofold one. This axis and the two mirror planes (also *C*_2_ axes) occur due to the equivalency of the trans pyrrolic C–N bonds (Figure 5). The Δ value (difference between shorter and longer N_meso_–C bonds) increases with the Pc negative charge when the number of F is constant, as observed for the [Cu^II^(F_16_Pc)^*n*−^]^(*n*−2)–^/[Cu^II^(F_8_Pc)^*n*−^]^(*n*−2)–^ (*n* = 3, 4) pairs.^28,29^ For *x* = 64, Δ increases from 0.014 Å for [Cu^II^(F_64_Pc^•3–^)]^−^ to 0.066–0.077 Å for [Cu^II^(F_64_Pc^4–^)]^2–^ (Table 2). Moreover, the Δ values for [Cu^II^(F_8_Pc)^*n*−^]^(*n*−2)–^ (*n* = 3, 4) are larger than those for [Cu^II^(F_16_Pc)^*n*−^]^(*n*−2)–^ and [Cu^II^(F_64_Pc)^*n*−^]^(*n*−2)–^ (*n* = 3, 4), consistent with the higher electronic deficiency of the F_16_Pc and F_64_Pc^19,20,23^ macrocycles in comparison with the F_8_Pc macrocycle. In general, the Pc macrocycles are more distorted in **2** and **3** relative to **1**.

A notable elongation of the C–F bonds was observed in a reduced Cu^II^(F_8_Pc) macrocycle, but those lengths were not affected by the reductions in Cu^II^(F_16_Pc).^28,29^ Cu^II^(F_64_Pc) has different types of C–F bonds, but all of them remained nearly unchanged by reductions.

The coordination environment of copper(II) centers in monoanionic copper(II) phthalocyanines {Cu^II^(F*_x_*Pc)^•3–^}^−^ (*x* = 8, 16, 64) was also analyzed by utilizing the SHAPE 2.1 software, which is based on the continuous shape measure (CShMs) algorithm of Pinsky and Avnir.^49^ The values given by the program tend to zero if the coordination sphere of a metal center forms a particular perfect reference polyhedron. Conversely, large values indicate strong deviations from the ideal geometry. In this case, we compare the observed geometry with an ideal square-planar geometry of copper(II) centers. The calculated coefficients are very small: 0.016 for *x* = 8, 0.000 for *x* = 16, and 0.005 for *x* = 64, indicating a nearly ideal square-planar geometry with *D*_4*h*_ symmetry in all three cases.

## Optical Properties

**Figure 6:**
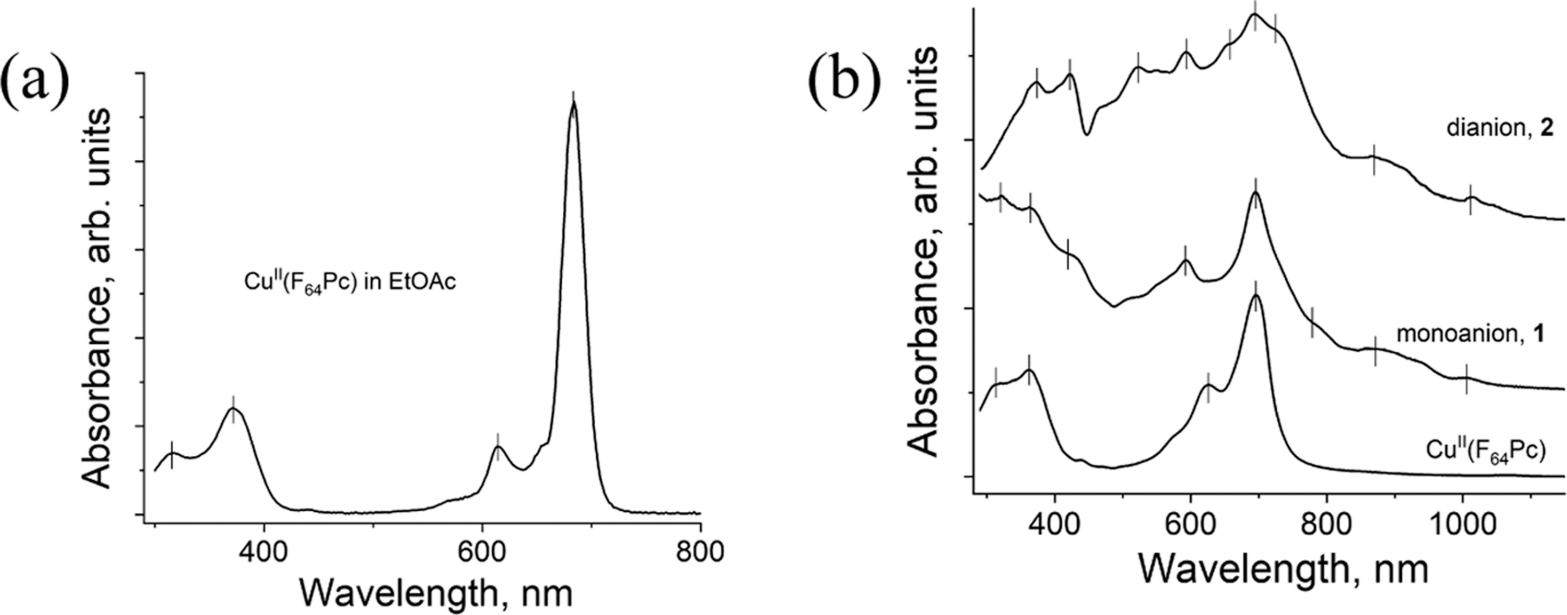
UV–vis–NIR spectra of Cu^II^(F_64_Pc) and its reduced species. (a) Solution spectrum of **0** in ethyl acetate and (b) solid-state spectra of Cu^II^(F_64_Pc), salts **1** and **2** in the KBr pellet. Vertical bars are used to mark spectral features. Absorbances are in arbitrary units. The absorption bands are listed in Table 3.

The UV–vis–near-infrared (NIR) spectra of parent Cu^II^(F_64_Pc) and anionic salts **1** and **2**, obtained using KBr pellets prepared under Ar, are shown in Figure 6.

The increase in the fluorine content results in bathochromic shifts, consistent with the data obtained in solution.^37^ Cu^II^(F_64_Pc^2–^)(C_4_H_8_O_2_)_2_ (**0**) exhibits Soret and Q-band maxima at 363 and 696 nm, respectively. For anions, the reduction of Cu^II^(F_8_Pc) and Cu^II^(F_16_Pc) was accompanied by a hypsochromic shift of the Q-bands.^28^ Cu^II^(F_64_Pc) behaves differently. The ligand-only reduction is most clearly defined in the Zn complex. Thus, (Cl^–^)[Zn(F_64_Pc^2–^)]^0^ exhibits strong bands at 430 and 692 nm in DMF. The photochemical reduction of this solution led to the gradual formation of the [Zn^II^(F_64_Pc^•3–^)]^−^ anions, with Q-band maxima at 468, 594, and 743 nm.^37,46^ The photochemical reduction of Zn(F_64_Pc) in ethanol,^16^ and electrochemical reduction of both Zn(F_64_Pc) and Cu(F_64_Pc) in trifluorotoluene^19^ had the same spectroscopic effect. In the case of **1**, the intensity of the 695 nm band decreases relative to Cu^II^(F_64_Pc), and new bands appear at 426, 592, and 777 nm (Figure 6 and Table 3). The same set of bands is observed in the spectrum of [Zn^II^(F_64_Pc^•3–^)]^−^, thus supporting the formation of F_64_Pc^•3–^. The spectroscopy of **2**, the doubly reduced [Cu^II^(F_64_Pc^4–^)]^2–^ is more complex, spectral data for the tetraanion (F_64_Pc)^4–^ being reported here for the first time. The Q-bands split in **2** giving rise to new bands in the visible region. Some splitting in the Soret region, as well as a hypsochromic shift, is also noted, effects that are similar to those observed for other Pc^4–^ tetraanions.^50^

**Table 3:** UV–vis–NIR Data of Fluorinated Phthalocyanines and Reduced Complexes^a^

		Absorption band λ_max_ [nm], sh: shoulder, m; most intense peak in the Q-band		
Compound	*n* in Pc^(2+*n*)–^	Soret band	Q-band	NIR
Cu^II^(F_8_Pc)^28^	0	332	612(m), 682, 735	1080 weak
{A}[Cu^II^(F_8_Pc^3•–^)]^−^ ^28^	1	317	574	821, 923, 1625 (CT)
{Bu_4_N^+^}_2_[Cu^II^(F_8_Pc^4–^)]^2–^·2solv^29^	2	319	590	1013
Cu^II^(F_16_Pc)^28^	0	339	628(m), 789	1137 weak
(PPN^+^)_3_[Cu(F_16_Pc)]_3_^3–^·2C_6_H_5_CN^29^	1	324	571	912, 1414 (CT)
{A}_2_[Cu^II^(F_16_Pc^4–^)]^2–^ ^28^	2	324	544(m), 623(sh)	833, 934, 1670 (CT)
Cu^II^(F_64_Pc^2–^)·2-ethyl acetate, **0**	0	319, 363	626, 696(m)	
Cu^II^(F_64_Pc) (in ethyl acetate), **0**	0	318, 372	614, 684(m)	
[Zn^II^(F_64_Pc^•3–^)·2-ethanol]^16^	1	452	452, 589, 695 729 (m)	924,1038
{B}[Cu^II^(F_64_Pc^•3–^)]^−^·2solv, **1**	1	320, 363, 426 (sh)	592, 695(m), 777(sh)	871, 1006
{B}_2_[Cu^II^(F_64_Pc^4–^)]^2–^·solv, **2**	2	373, 422	523, 594, 656(sh) 695(m), 720(sh)	868, 1005

a CT: charge-transfer band; A: cryptand(Na^+^); B: cryptand(K^+^); solv: C_6_H_4_Cl_2_; and PPN^+^: bis(triphenylphosphine)iminium.

Weak NIR absorptions of the radical anion and dianion are present at 868–871 and 1005–1006 nm, consistent with bands observed for all reduced phthalocyanines,^30,31,42,43,51^ including [Zn^II^(F_64_Pc^•3–^)]^−^,^16^ and tetrapyrazinoporphyrazine^42−45^ macrocycles. Such bands are more intense relative to those of **1** and **2**, but the bands for the related Cu^II^(F_8_Pc) and Cu^II^(F_16_Pc) are also weak (see Table 3).^28,29^

In addition to the Soret and Q-bands, charge-transfer (CT) bands are also noted. Such bands, usually broad, are caused by the π–π interaction between the macrocycles, in stacks or chains, as noted for the unhindered [Cu^II^(F*_x_*Pc)^*n*−^]^(*n*−2)–^ (*x* = 8, 16; *n* = 3, 4) anions,^28,29^ consistent with the tendency of fluorinated macrocycles with aromatic fluorine substituents to aggregate via π-stacking. However, the introduction of eight, bulky perfluoroisopropyl substituents, in the peripheral positions of Pc, led to molecular isolation of F_64_Pc macrocycles in M^II^F_64_Pc.^13,17,23,38^ Since the isolation in the solid state is enhanced upon reduction due to the presence of bulky cryptand(K^+^) counterions, CT bands beyond 1100 nm are not observed for any reduced F_64_Pc.

## Magnetic Interactions and F Effects

Relationships between the electronic and structural properties of reduced F_64_Pc species, especially given the possible simultaneous presence of unpaired electrons on both metal and ligand centers, are revealed by paramagnetic resonance and magnetic properties, summarized in Table 4.

**Table 4:** EPR and Magnetic Data (Superconducting Quantum Interference Device (SQUID)) for Reduced Cu^II^(F*_x_*Pc) Complexes and the Parent Nonreduced Complex, *x* = 64^a^

	Magnetics			EPR
Complex Cu:Pc spins	Magn. moment (μ_B_)	Weiss temp. (K)	Magnetic exchange interaction constant *J* (cm^–1^)	*A*_||_, Δ*H*: mT
{A}[Cu^II^(F_8_Pc^•3–^)]^−^ 1/2:1/2	2.39	– 9	intra-dimer *J*_R–R_ = −21.8	IS; HFI below 155 K; 298 K: *g* = 2.1652, Δ*H* = 95.02
			inter-dimer *J*_R–R_ = −14.6	
			*J*_Cu–R_ = −10.8, *J*_Cu–Cu_ = −1.5, *T* = max of magn. suscept. at 9 K	
{B}[Cu^II^(F_8_Pc^4–^)]^2–^·2solv 1/2:0	1.60	–4		298 K: *g*_||_ = 2.097, *A*_||_ = 26.56, *g*_⊥_ = 1.9628
{C}[Cu(F_16_Pc)]_3_^3–^·2C_6_H_5_CN pairs of [Cu^II^(F_16_Pc)^•3–^]^−^ separated by diamagnetic [Cu^I^(F_16_Pc)^**2**−^]^−^ ^28^	3.25	–21.5	*J*_inter_ = −16.3	an EPR signal was not observed
*S* = [(1/2 + 1/2) + (1/2 + 1/2)] separated by (0 + 0)			*J*_intra_ = −5.6	
{A}_2_[Cu^II^(F_16_Pc)^4–^]^2–^^29^ 1/2:0	1.95	–1		298 K: *g*_||_ = 2.1806, *A*_||_ = 20.11, *g*_⊥_ = 1.9597
				4.2 K: *g*_||_ = 2.1638, *A*_||_ = 21.66, *g*_⊥_ = 1.9586
Cu^II^(F_64_Pc^2–^)(C_4_H_8_O_2_)_2_, powder, **0** 1/2:0	spin only	0	0	4.5 K: *g*_||_ = 2.1650, *A*_||_ = 23.00, *g*_⊥_ = 2.036, *A*_⊥_ = 2.85
Cu^II^(F_64_Pc) in ethanol,^23^**0** 1/2:0				4.5 K: *g*_||_ = 2.1855, *A*_||_= 22.11, *g*_⊥_ = 2.0484, *A*_⊥_= 2.18
{D}[Cu^II^(F_64_Pc^•3–^)]^−^·2solv, **1** 1/2:1/2	2.55	–1	*J*_intra_ = −0.56	IS 298 K: *g*_1_ = 2.1723, Δ*H*_1_ = 33.2, *g*_2_ = 1.9514, Δ*H*_2_ = 80.4
{D}_2_[Cu^II^(F_64_Pc^4–^)]^2–^·solv, **2** 1/2:0				4.2 K: *g*_1_ = 2.1154, Δ*H*_1_ = 13.3, *g*_2_ = 2.0617, Δ*H*_2_ = 72.7

a {A}: {cryptand(Na^+^)}; {B}: (Bu_4_N^+^)_2_; {C}: (PPN^+^)_3_; {D}: {cryptand(K^+^)}; solv: C_6_H_4_Cl_2_; *J*_intra_/*k*_B_: Cu(II):Pc^•3–^ radical; *J*_inter_/*k*_B_= intermolecular coupling; HFI: hyperfine interaction of the unpaired electron on Cu^II^ atoms with the nuclear spin of ^63^Cu and ^65^Cu (*I* = 3/2); IS: intermediate signal of Cu^II^ and (F_8_Pc)^•3–^; R: radical.

Insights into the electronic structure of the reduced species, exhibiting unpaired spins located in both metal and ligand frontier orbitals, are provided by magnetic resonance. Information on the ligand-located spins was provided by the solution EPR of [Zn(F_64_Pc^•3–^)]^−^.^16,37,46^ The X-band CW–EPR spectrum of the ethanol solution of photo-reduced Zn^II^(F_64_Pc), the Zn analogous of Cu^II^(F_64_Pc), reveals a *g* = 2.003 broad signal, attributed to [Zn^II^(F_64_Pc^•3–^)]^−^, the Zn analog of **1**. The same signal is obtained upon chemical reduction. No intermolecular magnetic couplings are detected, an observation relevant to the magnetic interactions possible when diamagnetic Zn(II) is replaced by paramagnetic Cu(II). Conversely, magnetic resonance studies of Cu^II^(F_64_Pc) reveal the features of a spin located exclusively on the coordinated metal center, as opposed to exclusively on the Pc ring, like in the previous case.

X-band CW–EPR (Table 4) and ENDOR of solid, magnetically undiluted **0**, and its solutions in ethanol, established the noninteracting nature of the Cu spins. Hyperfine couplings have also been revealed. The results have been corroborated by density functional theory computations.^23^ Taken together, these findings regarding single-spin complexes are to be compared with cases when two spins are present simultaneously, i.e., reduced species.

The reduction of fluorinated Cu^II^(F*_x_*Pc) (*x* = 8, 16) phthalocyanines is mainly ring-centered, and accompanied by the formation of [Cu^II^(F*_x_*Pc)^•3–^]^−^ and [Cu^II^(F*_x_*Pc)^4–^]^2–^ anions.^28,29^

**Figure 7:**
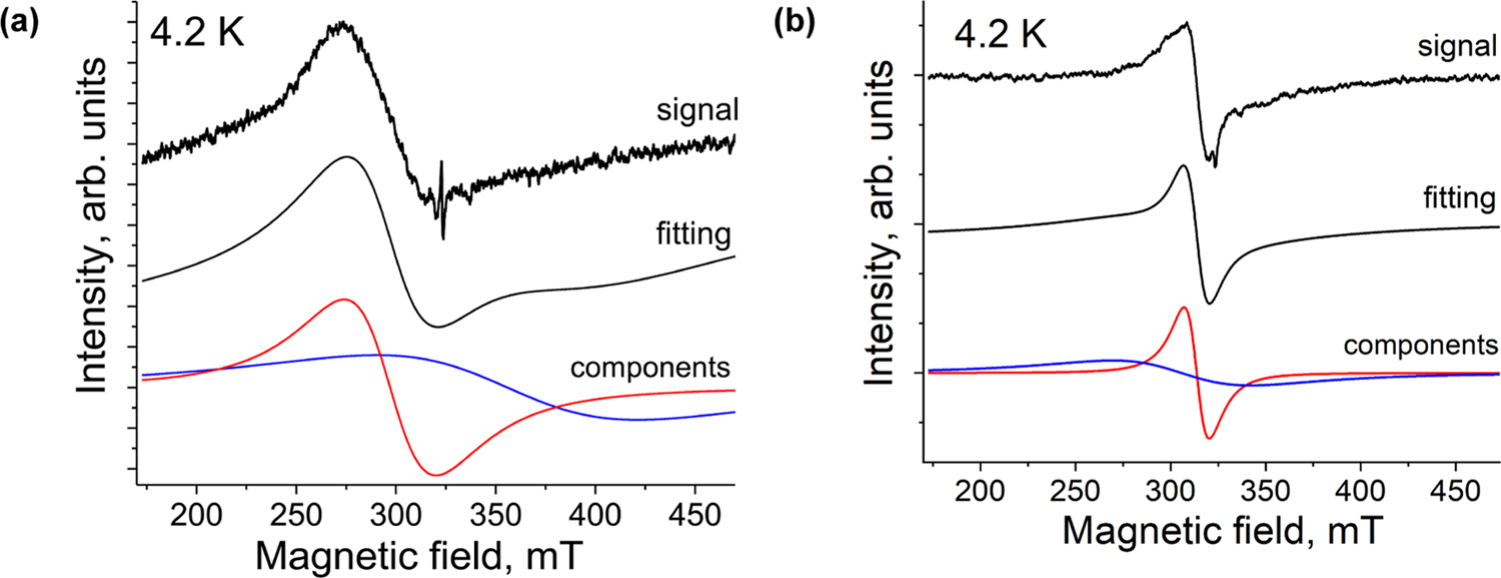
EPR spectra of polycrystalline (a) complex **1**; the *g* and Δ*H* components, *g*_1_ = 2.1769, Δ*H*_1_ = 46.1 mT, *g*_2_ = 1.8176, Δ*H*_2_ = 130.7 mT observed at 4.2 K were attributed to both Cu^II^ and F_64_Pc^•3–^; (b) complex **2**; the *g* and Δ*H* components, *g*_1_ = 2.1154, Δ*H*_1_ = 13.3 mT; *g*_2_ = 2.0617, Δ*H*_2_ = 72.7 mT at 4.2 K were attributed to Cu^II^.

The EPR spectra of **1** exhibit a broad, asymmetric signal, which can be fitted well by two broad Lorentzian lines (Figures 7a and S2). At 270 K, a signal with *g*_1_ = 2.2144 (Δ*H*_1_ = 30.3 mT) and *g*_2_ = 1.9949 (Δ*H*_2_ = 63.8 mT) is observed (Figure S2). This signal originates from paramagnetic Cu^II^ and F_64_Pc^•3–^ species. These signals are different from those originating from individual Cu^II^ and F_64_Pc^•3–^ species. Thus, according to the *g*-factor value, Cu^II^ has a larger contribution to the *g*_1_ line, but the hyperfine splitting (HFS) characteristic of isolated Cu^II^ is not observed. The *g*_2_ line has a large contribution from F_64_Pc^•3–^, but this line is too broad to be assigned to the isolated F_64_Pc^•3–^ species. These observations can be explained by the existence of a weak exchange interaction between the Cu^II^ and F_64_Pc^•3–^ species. The *g*-factor (Figure S3a) and the linewidth of the signal (Figure S3b) are strongly temperature-dependent, getting broader and smaller, respectively, as the temperature decreases. At 4.2 K, a signal with *g*_1_ = 2.1769 (Δ*H*_1_ = 46.1 mT) and *g*_2_ = 1.8176 (Δ*H*_2_ = 130.7 mT) is observed (Figure 7). These data also support a weak, antiferromagnetic coupling. Previously, a symmetric broad signal with *g* = 2.1652 (Δ*H* = 95.02 mT) at room temperature was observed for [Cu^II^(F_8_Pc^•3–^)]^−^ monoanions.^29^

The low-temperature EPR signal of **2**, for which the ligand is diamagnetic, can also be fitted well by two Lorentzian lines with *g*_1_ = 2.1154 (Δ*H*_1_ = 13.3 mT) and *g*_2_ = 2.0617 (Δ*H*_2_ = 72.7 mT) at 4.2 K (Figure 7b). The observed parameters are close to those of [Cu^II^(F*_x_*Pc)^4–^]^2–^ (*x* = 8, 16) anions with spins localized exclusively on the Cu^II^ ions. However, in these cases, a HFS is noted, unlike the signals of **2**, which lack HFS. The line with larger *g*_1_ could be attributed to the parallel component of the signal, while the line with smaller *g*_2_ could be attributed to the perpendicular component.^23,28^ However, in the absence of EPR measurement of oriented single crystals, these attributions remain tentative.

**Figure 8:**
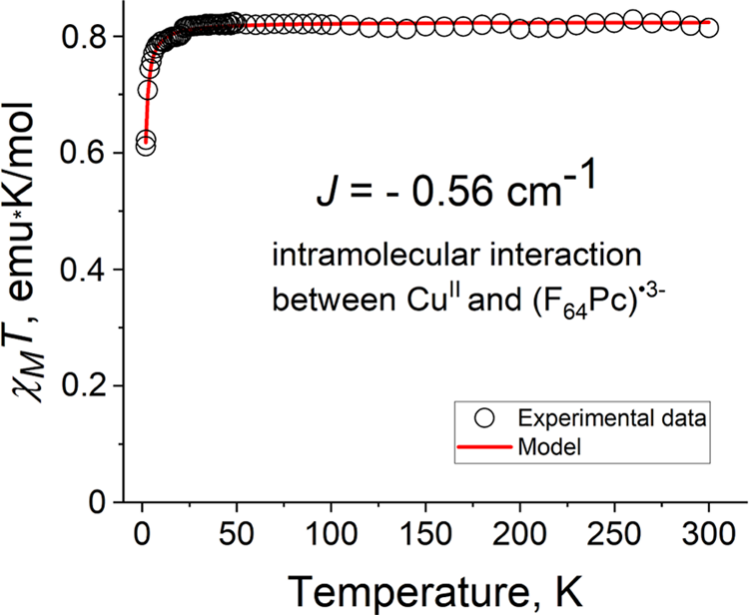
Temperature dependence of χ_M_*T* for **1** and its modeling.

Further insights into the electronic interactions are afforded by variable temperature static magnetic susceptibility measurements (Table 4 and Figure 8).

A spin-only value of the magnetic moment, in the absence of the Pc radical in **0**, results in a Curie paramagnet, i.e., Weiss temperature *Θ* = 0 K, indicating the absence of intermolecular Cu(II)–Cu(II) magnetic coupling.^23^ Magnetic moments close to the spin-only value of 1.73 μ_B_ are characteristic of the copper(II) complexes, but larger values up to 2.2 μ_B_ are attainable due to orbital contributions.^47,48^ In contrast to **0**, the effective magnetic moment of **1**, 2.55 μ_B_ (χ_M_*T* is 0.81 emu·K/mol) at 300 K (Figures 8 and S4a), is close to the value of 2.45 μ_B_ calculated for two noninteracting *S* = 1/2 spins and smaller than the 2.83 μ_B_ value expected for a triplet state.

The effective magnetic moment and χ_M_*T* value of **1** remain constant down to ∼20 K, and then decrease (Figures 8 and S4a). The temperature dependence of the reciprocal molar magnetic susceptibility (Figures 8 and S4) is linear over the whole temperature range. The observed Weiss temperature, *Θ* = −1 K, indicates a weak antiferromagnetic spin coupling.

The magnetic behavior of **1** was modeled using the PHI program^52^ to estimate the degree of magnetic coupling. The shortest Cu–Cu distance, ∼7.5 Å, is too long from a magnetic point of view for a reasonable intermolecular, through-space coupling, leaving the intramolecular coupling between the F_64_Pc^•3–^ radical and the d^9^ electron to be the dominant interaction. A good fitting of the experimental data (Figure 8) using *g*(Cu^II^) = 2.19 and *g =* 2.0000 for F_64_Pc^•3–^ yielded *J* = −0.56 cm^–1^. The ligand-only *g =* 2.0000 value is consistent with *g* = 2.003–2.009 measured for the solution, ligand-only signal of [Zn(F_64_Pc^•3–^)]^−^.^16,44^ Compared with F_64_Pc^•3–^, the intramolecular couplings between Cu^II^ and F*_x_*Pc^•3–^, *x* = 8 and 16, namely, −10.8 and −5.6 cm^–1^, respectively (Table 4) are at least 1 order of magnitude larger.^28,29^ As the degree of fluorination increases, the intramolecular magnetic coupling decreases, a trend consistent with the notion of enhanced spin density transfer to acceptor fluoro-substituents as *x* increases. Thus, the weakest coupling in [Cu^II^(F_64_Pc)^•3–^]^−^ could be explained by the extreme electron deficiency of its Pc core.^20,23^ The geometry around the copper(II) atoms most probably does not affected the *J* values since, according to the SHAPE 2.1,^49^ estimation, for all three anions with *x* = 8, 16, and 64, the geometry of copper(II) is very close to square-planar, *D*_4*h*_ symmetry. As a control experiment, the possibility of inter-Pcs coupling, mediated by the π-stacked solvents, was considered. Thus, the magnetic coupling was modeled with two *J*’s, but values with reasonable accuracy could not be obtained. *J* is too small for such manipulations.

In summary, magnetic measurements confirm the existence of magnetically isolated molecules, but subject to intramolecular spin coupling that is the lowest among the copper F*_x_*Pc^•3–^ species, as per an inverse correlation between its magnitude and *x*.

## Conclusions

New, crystalline salts based on 1- and 2-electron reductions of copper(II) 1,4,8,11,15,18,22,25-octafluoro-2,3,9,10,16,17,23,24-octakisperfluoro(isopropyl) phthalocyanine (Cu^II^F_64_Pc) were obtained, and their crystal and molecular structures were elucidated. There are no intermolecular π–π stacking interactions present, but such interactions are present between the Pc and the *o*-dichlorobenzene solvent of crystallization. The ligands are slightly twisted and form domes.

The Cu–N bonds are similar, and so are the benzene, pyrrolic, and all C–F bonds, with small variations. The major effect of reductions is the variation of N_meso_–C bonds, bonds that are equal in Cu^II^(F*_x_*Pc) except for *x* = 8. The reduced macrocycles exhibit alternative shortening and elongations of these bonds, resulting in the reduction of the fourfold axis to a twofold one.

This effect, linked to the partial loss of aromaticity when 19 and 20 π-electron complexes are formed, is more pronounced for the dianions [Cu^II^(F_64_Pc^4–^)]^2–^ vs monoanions [Cu^II^(F_64_Pc^•3–^)]^−^. Anionic species of Cu^II^(F_64_Pc) and Cu^II^(F_16_Pc), which contain highly electron-deficient Pc ligands, exhibit a smaller departure from bond equivalency and alternation in comparison with Cu^II^(F_8_Pc), which has a relatively diminished electronic deficient Pc ligand.

Structural, optical, and magnetic studies reveal that the reduction along the series [Cu^II^(F*_x_*Pc)^•3–^]^−^ and [Cu^II^(F*_x_*Pc)^4–^]^2–^ (*x* = 8, 16, 64) mono- and dianions is mainly localized on the macrocycle. The only exception is (PPN^+^)_3_[Cu(F_16_Pc)]_3_^3–^·2C_6_H_5_CN complex, in which both the reduced macrocycle [Cu^II^(F_16_Pc)^•3–^]^−^ and the reduced metal center [Cu^I^(F_16_Pc)^2–^]^−^species co-exist.^29^

New, weak bands appear in the NIR, while bands in the visible region shift and split. The first reduction leads to [Cu^II^(F*_x_*Pc^•3–^)]^−^ species with two *S* = 1/2 spins localized on Cu^II^ atoms and delocalized over the F*_x_*Pc macrocycle. Coupling between these two spins decreases in order [Cu^II^(F_8_Pc^•3–^)]^−^, [Cu^II^(F_16_Pc^•3–^)]^−^, and [Cu^II^(F_64_Pc^•3–^)]^−^ when the number of fluorine atoms increases. Most probably, the electron-withdrawing substituents have a crucial effect in stabilizing the anions. Monoanions [Cu^II^(F*_x_*Pc)^•3–^]^−^ (*x* = 8, 16) with fluorine atoms attached directly to the Pc core form mainly closely packed π-stacks. The [Cu^II^(F_64_Pc)^•3–^]^−^ anions are isolated due to the presence of bulky perfluoroisopropyl substituents on the periphery that completely suppress any intermolecular interactions. The [Cu^II^(F*_x_*Pc)^4–^]^2–^ (*x* = 8, 16, 64) dianions contain diamagnetic and EPR silent tetraanion macrocycles and paramagnetic Cu^II^ atoms with *S* = 1/2 spin state.

This work showed that [Cu^II^(F_64_Pc^2–^)] could be a promising, solution-processable acceptor component in constructing air-stable donor–acceptor assemblies, including via strong π–π stacking interactions with small donors. As such, the biradical nature of its monoanion complexes constitutes an attractive target for designing robust, magnetic, and probably conducting materials.

## Experimental Section

### Materials and Methods

Copper(II) 1,4,8,11,15,18,22,25-octafluoro-2,3,9,10,16,17,23,24-octakisperfluoro(isopropyl) phthalocyanine {Cu^II^(F_64_Pc)} was obtained as described.^23^ 4,7,13,16,21,24-Hexaoxa-1,10-diazabicyclo[8.8.8]hexacosane (cryptand, Acros Organics) and potassium graphite (KC_8_, Strem) were used as received. Solvents were purified under an argon atmosphere. *o*-Dichlorobenzene (C_6_H_4_Cl_2_) was distilled over CaH_2_ under reduced pressure; *n*-hexane was distilled over Na/benzophenone. The solvents were degassed and stored in a glovebox that contained less than 1 ppm of water and oxygen. The crystals of **1**–**3** were stored in the glovebox. KBr pellets for IR- and UV–vis–NIR measurements were also prepared in the glovebox. EPR (**1** and **2**) and SQUID measurements (**1**) were performed on polycrystalline samples sealed in 2 mm quartz tubes under an ambient pressure of argon gas.

### Syntheses and Crystallization of **1**–**3**

The salt {cryptand(K^+^)}[Cu^II^(F_64_Pc^•3–^)]^−^·2C_6_H_4_Cl_2_ (**1**) was obtained via the reduction of Cu^II^(F_64_Pc) (25 mg, 0.012 mmol) in the presence of one equivalent of cryptand (4.8 mg, 0.012 mmol) and an excess of potassium graphite (20 mg, 0.148 mmol) at 60 °C in 16 mL of *o*-dichlorobenzene and stirred for 24 h, under inert atmosphere.

The deep-blue solution formed was cooled to 25 °C, and potassium graphite was removed by filtering the solution into a 50 mL glass tube of 1.8 cm diameter with a ground glass plug, followed by the careful layering of 30 mL of *n*-hexane. Large, black prismatic crystals, formed after 1–2 months in a 20% yield, were washed with *n*-hexane. No other types of crystals formed, as revealed by the microscopic examination of crystals and unit cell determinations for several specimens. Calculated for C_86_H_44_Cl_4_N_10_O_6_F_64_KCu: C, 37.21; H, 1.59; N, 5.05. Found: C, 36.89; H, 2.01; N, 5.19.

The salt {cryptand(K^+^)}_2_[Cu^II^(F_64_Pc^4–^)]^2–^·C_6_H_4_Cl_2_ (**2**) was obtained via the reduction of Cu^II^(F_64_Pc) (25 mg, 0.012 mmol) in the presence of one equivalent of cryptand (4.8 mg, 0.012 mmol) and using an excess of potassium graphite (20 mg, 0.148 mmol) at 60 °C in 16 mL of *o*-dichlorobenzene, under inert atmosphere. After the deep-blue solution was stirred for 24 h, one more equivalent of cryptand (4.8 mg, 0.012 mmol) and an excess of potassium graphite (20 mg, 0.148 mmol) were added. The solution turned red-purple while stirred for an additional 24 h. The mixture was cooled to room temperature, and the potassium graphite was filtered out. The clear solution was placed in a glass tube and carefully layered with *n*-hexane. After 1 month, several large crystals having the shape of elongated plates and weighing about 2 mg formed on the walls of the tube. A small amount of powder also formed at the bottom of the tube. The crystals were removed from the solvent via decanting, washed with several portions of *n*-hexane, and the solvent was decanted every time to remove any powder to yield pure crystals. Four large crystals were examined via X-ray diffraction, and the unit cell of **2** was confirmed in each case. These crystals were used for nondestructive EPR studies and for the determination of optical properties of **2**.

The crystals of {cryptand(K^+^)}[Cu^II^(F_64_Pc)^•3–^]^−^ (**3**) were obtained via the reduction of Cu^II^(F_64_Pc) (25 mg, 0.012 mmol) in the presence of two equivalents of cryptand (9.6 mg, 0.024 mmol) and an excess of potassium graphite (20 mg, 0.148 mmol) at 60 °C in 16 mL of *o*-dichlorobenzene upon stirring for 24 h under inert atmosphere. The mixture was cooled to room temperature, and the potassium graphite was filtered out. The clear solution was placed in a glass tube and carefully layered with *n*-hexane. Crystals formed after 1 month. The crystals were isolated by decanting the solvents and subsequently washed with several portions of *n*-hexane. Most crystals were large prisms with the cell parameters of **1**. Several small black plates, easily distinguishable from the large prisms under microscopic examination, were separated manually (Pasteur method). All plates were tested by X-ray diffraction and shown to belong to only one crystal phase with the cell parameters of **3**.

#### General Methods

##### UV–vis–NIR, EPR and SQUID Magnetic Measurements

UV–vis–NIR spectra were measured in KBr pellets on a PerkinElmer Lambda 1050 spectrometer in the 250–2500 nm range. EPR spectra were recorded for the polycrystalline samples of **1** and **2** with a JEOL JES-TE 200 X-band ESR spectrometer equipped with a JEOL ES-CT470 cryostat. A Quantum Design MPMS-XL SQUID magnetometer was used to measure the static magnetic susceptibility of **1** at a 1 kOe magnetic field in cooling and heating conditions in the 300–1.9 K range. The sample-holder contribution and core temperature-independent diamagnetic susceptibility (χ_d_) were subtracted from the experimental values. The χ_d_ values were estimated from the extrapolation of the data in the high-temperature range by fitting the data with the following expression: χ_M_ = *C*/(*T* – *Θ*) + χ_d_, where *C* is the Curie constant and *Θ* is the Weiss temperature. Effective magnetic moments (μ_eff_) were calculated with the formula μ_eff_ = (8χ_M_*T*)^1/2^.

### X-ray Crystal Structure Determination and Analyses

X-ray diffraction data for **1**–**3** are listed in Table 5. The X-ray diffraction data for complexes **1**–**3** were obtained on a D8 Venture diffractometer (Bruker, Germany) in the ϕ- and ω-scanning modes at the center of the shared equipment, Kurnakov Institute of General and Inorganic Chemistry, Russian Academy of Sciences (λ = 0.71073 Å, Incoatec IμS 3.0 microfocus X-ray source). Primary indexing, the refinement of unit cell parameters, and the integration of reflections were performed using the Bruker APEX3 software package.^53^ The reflection intensities were corrected for absorption using the SADABS software.^54^ The structures were solved by direct methods^55^ and refined by the full-matrix least-squares techniques against *F*^2^ using the program suite.^56,57^

**Table 5:** X-ray Diffraction Data for **1**–**3**

compound	**1**	**2**	**3**
emp. formula	C_86_H_44_Cl_4_CuF_64_KN_10_O_6_	C_92_H_72_CuF_64_K_2_N_12_O_12_ (+ squeezed one C_6_H_4_Cl_2_ molecule)	C_92_H_72_CuF_64_K_2_N_12_O_12_
*M*_r_ (g/mol)	2773.75	2895.35	2895.35
color and shape	black prism	black, prismatic	black plate
crystal system	triclinic	triclinic	triclinic
space group	*P*1̅	*P*1̅	*P*1̅
*a* (Å)	17.0999(8)	16.3374(5)	13.3077(5)
*b* (Å)	18.2638(8)	19.0573(6)	15.1963(5)
*c* (Å)	19.3623(9)	20.9513(7)	15.2534(5)
α (deg)	113.1521(16)	78.7461(12)	70.5040(12)
β (deg)	111.3183(16)	79.0264(11)	83.4840(13)
γ (deg)	95.6638(17)	68.6512(11)	71.3761(13)
*V* (Å^3^)	4969.2(4)	5907.6(3)	2755.45(17)
*Z*	2	2	1
ρ_calc_ (g/cm^3^)	1.854	1.628	1.745
μ (mm^–1^)	0.549	0.417	0.447
*F*(000)	2740	2894	1447
*T* (K)	100(2)	100(2)	100(2)
max. 2Θ (deg)	50.118	51.456	57.314
reflns measured	69 600	80 229	40 239
unique reflns	17 620	22 486	14 169
parameters	1549	1871	852
restraints	0	73	15
reflns[*F*_o_ > 2(*F*_o_)]	13 440	16 114	11 432
*R*_1_[*F*_o_ > 2σ(*F*_o_)]	0.0602	0.0643	0.0388
W*R*_2_ (all data)	0.1246	0.1780	0.1018
G.O.F.	1.048	1.053	1.043
CCDC number	2238185	2238187	2238186

Nonhydrogen atoms were refined in the anisotropic approximation, except for some fluorine and carbon atoms in disordered parts of trifluoromethyl groups. Equivalent fluorine–carbon distances in these molecular fragments were restrained to be approximately equal by applying SADI restraints, as well as thermal displacement parameters were restrained by using the SIMU instruction at the refinement. All hydrogen atoms were placed in calculated positions, riding on the nonhydrogen atoms to which they were bonded. Three perfluoroisopropyl groups are disordered between two orientations with 0.650(3)/0.350(3), 0.650(3)/0.350(3), and 0.803(5)/0.197(5) occupancies in **2**. In the crystal structure of **2**, there is one highly disordered *o*-dichlorobenzene molecule, one molecule of solvent is present per unit cell, which was squeezed using the standard Olex2/PLATON routine.^58^ One −CH_2_–CH_2_–O–CH_2_–CH_2_–O–CH_2_–CH_2_– group of cryptand(K^+^) cation and the fluorine atoms of one CF_3_ group are disordered over two orientations with 0.925(5)/0.075(5) and 0.930(4)/0.070(4) occupancies in **3**. Structural visualizations, aromaticity calculations, and intermolecular interactions have been obtained using the Mercury package.^39^

## Supporting Information Available

Packing diagrams, EPR, magnetics, and π interactions for new compounds (PDF)
