# Supplementary material for: Synthesis, X-ray Structures, and Optical and Magnetic Properties of Cu(II) Octafluoro-octakisperfluoro(isopropyl)phthalocyanine: The Effects of Electron Addition and Fluorine Accretion
Source: Inorg Chem. 2023 Jul 12;62(29):11390–401. doi: 10.1021/acs.inorgchem.3c00887 (PMC10369491; doi:10.1021/acs.inorgchem.3c00887)
Supplement: Supplementary file 1 — ic3c00887_si_001.pdf [file ic3c00887_si_001.pdf]

Supporting information

Synthesis, X-ray structures, and optical and magnetic properties of Cu(II) octafluoro-octakisperfluoro(isopropyl)phthalocyanine: the effects of electrons addition and fluorine accretion

Maxim A. Faraonov,[a]* Ilya A. Yakushev,[b] Evgeniya I. Yudanova,[a] Marius Pelmuş,[c] Sergiu M. Gorun,[c*] Akihiro Otsuka,[d] Hideki Yamochi,[d] Hiroshi Kitagawa,[d] and Dmitri V. Konarev[a]*

[a]Federal Research Center of Problems of Chemical Physics and Medical Chemistry RAS, Chernogolovka, Moscow region, 142432, Russia.

[b]Kurnakov Institute of General and Inorganic Chemistry, Russian Academy of Sciences, Moscow, 119991, Russia.

[c]Department of Chemistry and Biochemistry and Center for Functional Materials, Seton Hall University, South Orange, NJ 07079, USA.

[d]Division of Chemistry, Graduate School of Science, Kyoto University, Sakyo-ku, Kyoto 606-8502, Japan.

* Corresponding authors email:
maksimfaraonov@yandex.ru
sergiu.gorun@shu.edu
konarev3@yandex.ru

Table of contents

Page

Figure S1. Packing diagrams of **1**, **2**, and **3**…………………………………..……………….S3

Figure S2. EPR spectra of polycrystalline **1**……..……,………………………..…………….S4

Figure S3. Temperature dependence of EPR parameters of **1.**…………………….. …………..S4

Figure S4. Temperature dependence of the magnetic moment and susceptibility of **1**………S4

Figure S5. Intermolecular, Pc phenyl - *o*-chlorobenzene rings π-π interactions in **1**……………S4

Figure S6. Conformation analysis of *i*-C_3F_7 groups……………………………………………..S5

Table S1. Aromaticity parameters of solvent-Pc interactions **1**……………...………..………...S5

References………………………………………………………………………………….S6

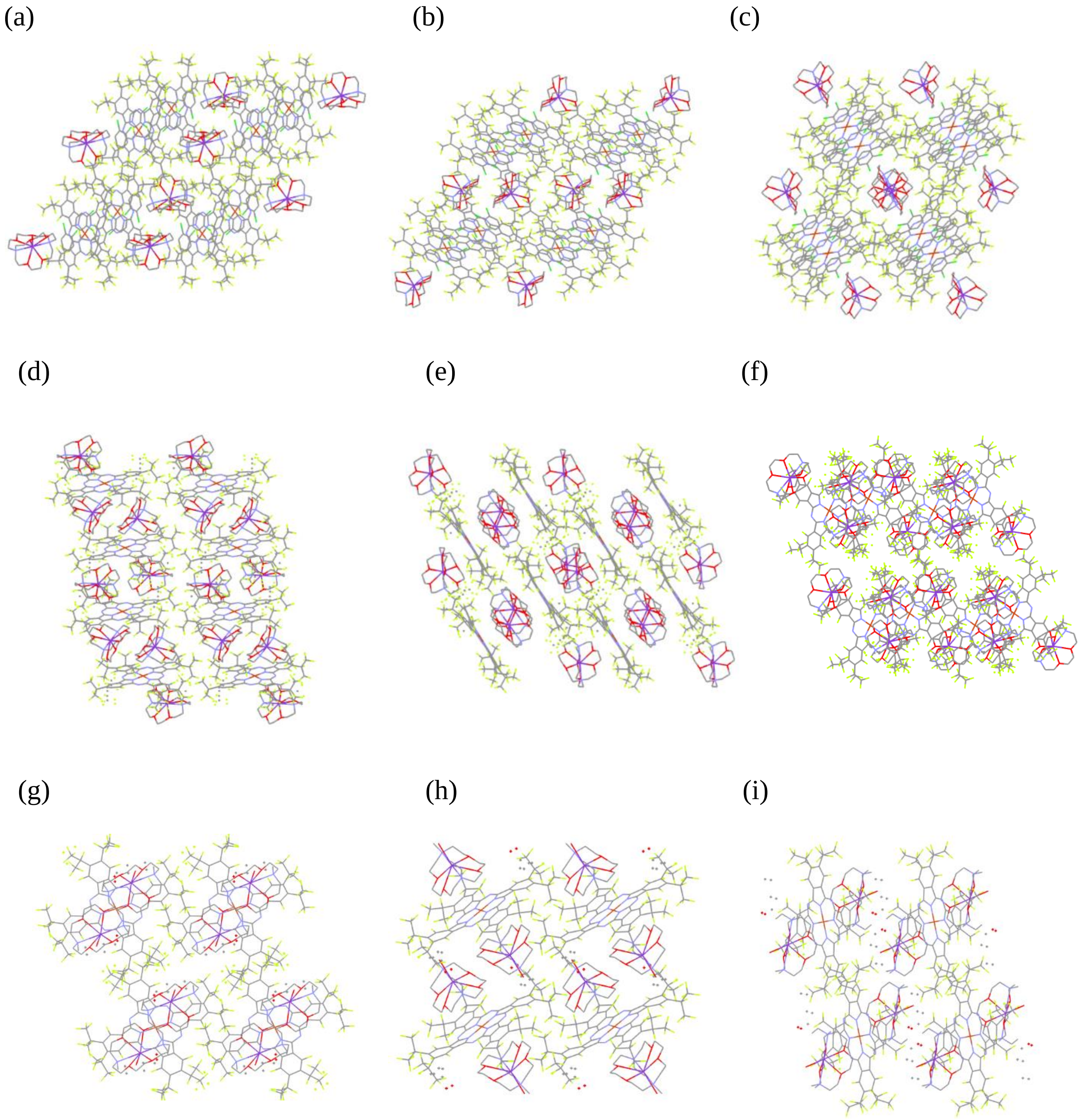

Figure S1. Packing diagrams of two unit cells of **1**: [(a), (b), (c)], **2**: [(d), (e), (f)], and **3**: [(g), (h), (i)], viewed along the *a*, *b*, and *c* crystallographic axes, respectively. The hydrogen atoms have been omitted for the sake of clarity.

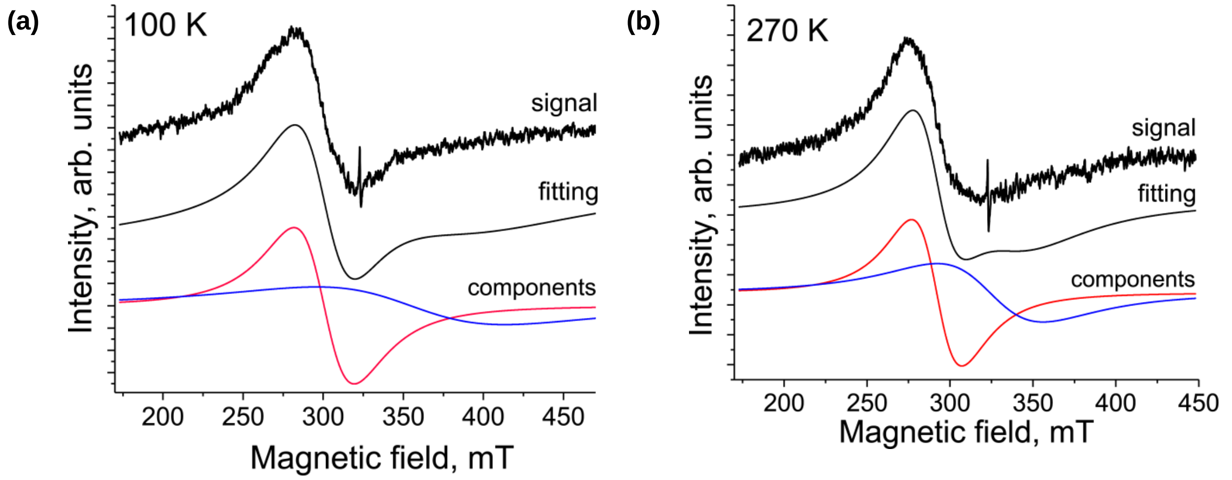

Figure S2. EPR spectra of polycrystalline **1** at (a) 100 K, and (b) 270 K.

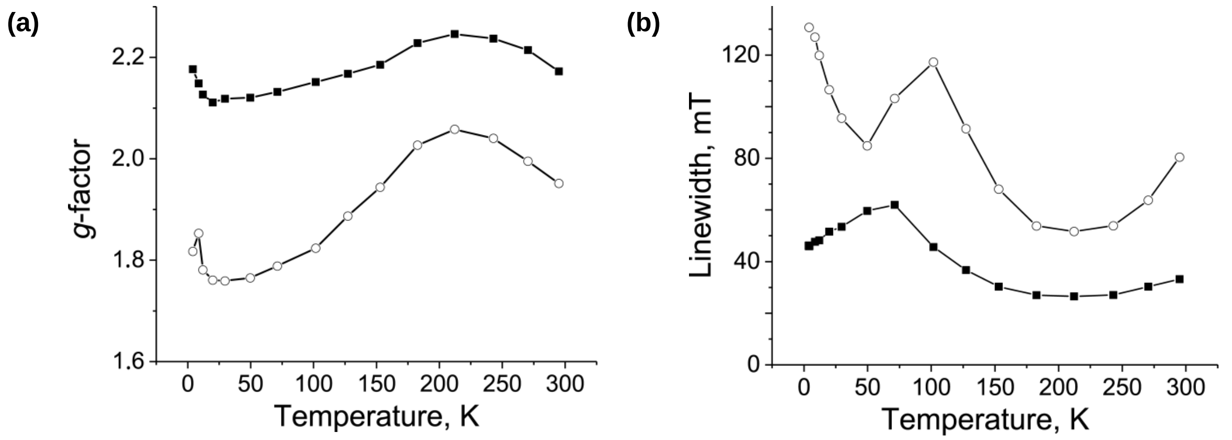

Figure S3. Temperature dependence of (a) the *g*-factor, and (b) the linewidth of the *g* components of the EPR signals of **1.**

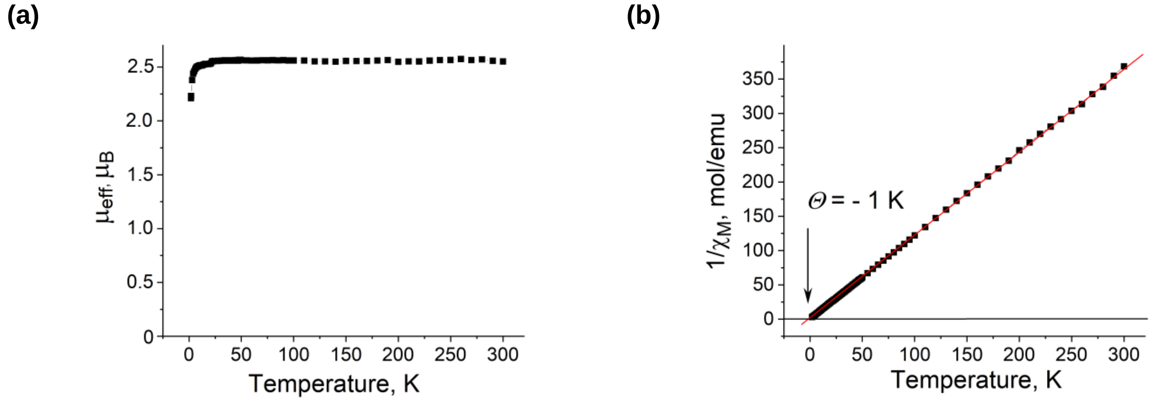

Figure S4. Temperature dependence of (a) the effective magnetic moment, and (b) the reciprocal molar magnetic susceptibility of **1**.

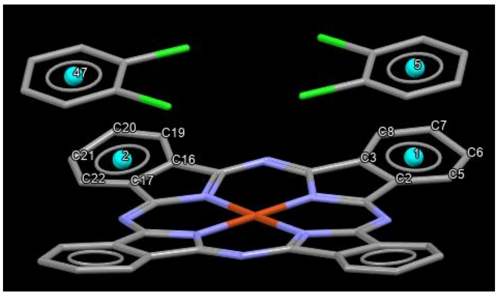

Figure S5. Intermolecular π-π interactions between the Pc benzene rings (centroid 1 and 2) and the 2 *o*-dichlorobenzene molecules, centroids labeled # 5 and 47, respectively in the crystal structure of **1**. Similar interactions occur with the other Pc molecules of the "dimer", see Figure 4a.

Table S1. Aromaticity interaction strengths evaluation and inter-centroids distances, and the angles between *o*-dichlorobenzene and Pc rings in **1**, computed using a literature procedure.[1] Similar interactions occur between the solvents and the other Pc of the "dimer", Figure 4a.

Centroid1	Centroid2	Distance (Å)	Relative solvent-Pc rings orientations (°)	Score[a]	Assessment
1	5	3.90	7.4	9.2	Strong
2	47	4.06	5.5	8.9	Strong

[a] Scores between 7 and 10 indicate strong π-π interactions.

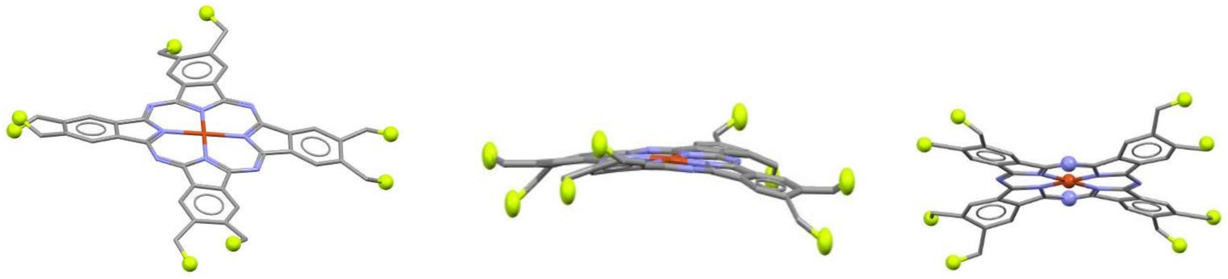

(a) (b) (c)

Figure S6. The conformation of ortho *i*-C_3F_7 groups, as revealed by the position of the tertiary F above or below the Pc mean plane. There are 4 phenyl moieties, each bearing 2 ortho *i*-C_3F_7 groups, whose sterically encumbered tertiary F groups could be located either above, (A), or below, (B), the mean plane of the Pc ring, defining, therefore AA, AB and BB conformational combinations. The F groups, attached to sp^3 hybridized C atoms, are too close to be both located in the plane of the phenyl rings. Viewing the molecules along the Pc mean plane, and circling around the Pc ring anti-clockwise, each F will appear, *in turn*, in an A or B position. Thus, each Pc will be labelled, for example, (AB)(AB)(BA)(AB), to indicate in this case that, the F are located

above-below the plane for the first 2 phenyl groups, followed by below-above for the 3rd phenyl group, and, again, above-below for the 4th phenyl group. The starting point is irrelevant.

(a) Molecule **1:** (BA)(BA)(BA)(AB). (b) Molecule **2:** (BA)(BA)(BA)(BA). This conformation exhibits a C_4 axis perpendicular to the Pc plane. (c) Molecule **3:** (BA)(BA)(AB)(AB). This conformation is palindromic and thus exhibits a C_2 axis perpendicular to the Pc plane and 2 mirror planes; one, for example, incorporates the N-Cu-N atoms depicted as ball-and-stick; both orthogonal to the Pc ring, in between the neighboring AA and BB groups . The C_4 axis is lost.

References

1. Macrae, C. F.; Sovago, I.; Cottrell, S. J.; Galek, P. T. A.; McCabe, P.; Pidcock, E.; Platings, M.; Shields, G. P.; Stevens, J. S.; Towler, M.; Wood, P. A. Mercury 4.0: From Visualization to Analysis, Design and Prediction. *J. Appl. Cryst.* **2020**, *53*, 226–235. https://doi.org/10.1107/s1600576719014092.
